# Solid-state platform (SSP) to produce terpenes from enzymatically treated textile waste with *E. coli*

**DOI:** 10.1039/d5ra06351e

**Published:** 2026-01-14

**Authors:** Žiga Zebec, Vid K. Bučar, Brigita Hočevar, Mojca Poberžnik, Miha Grilc, Blaž Likozar, Aleksandra Lobnik

**Affiliations:** a Institute for Sensors and Environmental Protection, IOS d.o.o Maribor Slovenia; b University of Maribor, Faculty of Mechanical Engineering Maribor Slovenia aleksandra.lobnik@um.si; c Institute of Informational Science Maribor, IZUM Slovenia ziga.zebec@izum.si; d National Institute of Chemistry Ljubljana Slovenia

## Abstract

Recycling waste has become one of the critical issues facing modern societies, with textile waste streams serving as a primary example for the recycling of materials made from cotton, PET, other plastics, and their mixtures. One method that has gained traction in recent years is enzymatic hydrolysis of textile waste and the subsequent conversion of cotton to glucose. Glucose serves as the carbon source for engineered microbes that convert it to volatile value-added chemicals such as terpenes. In this work, we present a novel strategy to produce volatile organic compounds (VOCs), the ‘Solid-State Platform’ (SSP) for the microbial synthesis of terpenes. This platform offers a sustainable approach for utilising enzymatically treated cotton-based waste and its efficient transformation by engineered *Escherichia coli* into VOCs, which are trapped directly as vapour by the organic overlay, dodecane, present within the SSP. The engineered microbial system is constructed from conventional inert laboratory glass, without gaskets, filters, or high-tech custom-made equipment. Using pure glucose as the carbon source 1.4 mg mL^−1^ of limonene and 0.7 mg mL^−1^ cineole was produced respectively using the SSP. Using enzymatically treated waste materials, specificaly glucose juice H46, 0.35 mg mL^−1^ of cineole was produced by SSP. The SSP represents an alternative strategy for producing green volatile value-added chemicals and may help mitigate the environmental burden of textile disposal by broadening the assortment of recycling technologies.

## Abstract 2

The Solid-State Platform (SSP) enables microbial production and direct capture of limonene or cineole as vapour in the product container, eliminating the need to remove residual cell debris and growth medium.

## Graphical abstract

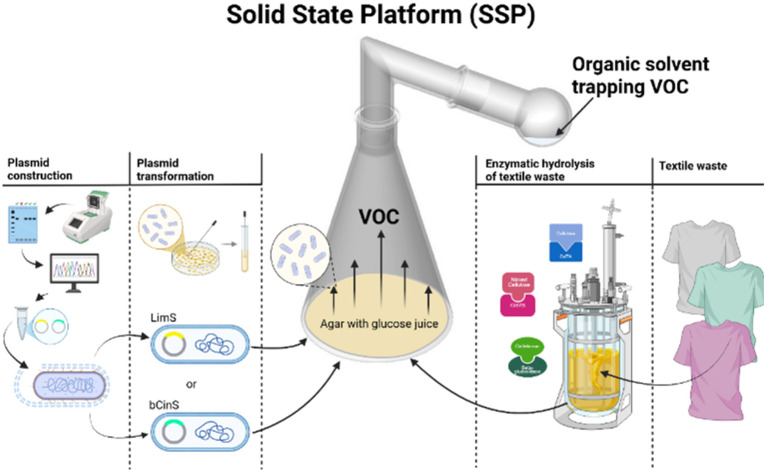

## Introduction

Textile waste is one of the most pressing challenges of modern industrialization, with an estimated 92 million tonnes generated globally each year and only 12% recycled.^1^ The remaining majority ends up in landfills or incineration, contributing significantly to environmental degradation and greenhouse gas emissions.^2^ The urgency of addressing this crisis is underscored by studies highlighting inefficiencies in current waste management systems, where advanced recycling technologies remain underutilized.^3^

Among the most impactful solutions, mechanical recycling has emerged as a cornerstone strategy, transforming cotton and linen waste into premium yarns and fabrics, effectively closing the loop and reducing reliance on virgin materials.^4^ Mechanically recycled textiles can reduce energy use by 50% and water consumption by up to 70% compared to virgin production. Similarly, chemical recycling methods, such as the depolymerization of polyester, recover essential monomers for high quality polymer production, setting a scalable precedent for industrial applications.^5,6^ Biobased innovations, including bacterial cellulose derived from mixed textile and agricultural waste, demonstrate the convergence of natural processes with industrial needs and offer the potential to process 35% of currently non-recyclable textile waste.^7^

Composting initiatives represent a transformative approach in textile waste management, offering a sustainable solution for natural fibre recycling. By leveraging green chemistry principles, these efforts repurpose up to 15% of textile waste, significantly reducing landfill dependency while producing nutrient rich compost.^8^ Concurrently, advancements in dye effluent treatment recover essential resources such as dyes and salts, achieving impressive recovery rates of up to 80%, underscoring the synergy between environmental sustainability and economic viability.^9^ These innovations not only address textile disposal challenges but also unlock the potential to reuse millions of tonnes of cotton waste annually, enhancing the market value of recycled products and fostering the circular textile economy.^10^ Lifecycle analyses further demonstrates the environmental benefits of these approaches, showing reductions in the carbon footprint of textile recycling by 30–50%, depending on the processing method and material.^11^ However, the global nature of textile production and waste generation necessitates localized solutions, it also highlights the importance of addressing stark geographical disparities in recycling efficiency.^12^

Enzymatic hydrolysis offers a biocatalytic route to depolymerize waste into reusable chemicals such as cellulose nanocrystals,^13^ combining efficiency with a low environmental footprint and achieving over 85% conversion of polyester-cotton blends.^14^ Unlike conventional methods, enzymatic processes avoid toxic solvents and selectively target polymers in mixed waste blends.^15^ While enzyme costs, kinetics, and pre-treatment requirements hinder scalability,^16^ advances in protein engineering could position this waste valorisation technology as a linchpin for a circular textile economy.^17^

One strategy to valorise textile waste is the production of ‘value added chemicals’ of which some are also volatile (VOCs) like terpenes, such as limonene and cineole. These natural products, synthesized by a wide range of plants are playing key roles as ecological signal molecules that regulate various interactions. Limonene, a dominant monoterpene found in citrus peels, can constitute up to 71% of the volatile profile in some plant families and exhibits notable antifungal and antibacterial properties.^18^ Similarly, cineole (1,8-cineole), prominent in eucalyptus oil, is recognized for its distinctive camphoraceous aroma and widespread use in pharmaceuticals due to its antimicrobial efficacy.^19^ These compounds are typically biosynthesized through the mevalonate pathway, highlighting their metabolic efficiency and ecological significance. Advances in analytical techniques such as GC-MS (Gas Chromatography-Mass Spectrometry) have expanded our understanding of the composition and variability of these VOCs, revealing their prevalence in medicinal plants like *Taraxacum officinale* and their potential for diverse industrial applications.^20^ The versatility of terpenes such as limonene and cineole^21^ lies in their role as bioactive building blocks, valuable end-products or intermediaries for subsequent conversion to more advanced biomolecules.^22^

Recent advancements in metabolic engineering and synthetic biology have achieved a notable improvement in limonene production titter to 1.29 g L^−1^ by co-expressing optimized mevalonate pathway enzymes and a neryl pyrophosphate synthase, followed by fed-batch fermentation over 84 hours.^23^ Another approach aims to rewire the central carbon metabolism of *E. coli*, by applying a modular overexpression to the methylerythritol phosphate (MEP) pathway and controlling the competitive reaction (*e.g.* knockout) flux of the precursor molecule geranyl diphosphate (GPP), resulting in increased limonene yield.^24^ Limonene has been produced recently by our group also from enzymatic hydrolysis textile waste.^25^ In a fed-batch two-phase bioreactor setting, using the Limonene synthase (LimS) from pJBEI-6410 a titter of 3.6 g L^−1^ was reported in *E. coli*, which is the highest titter reported to our knowledge.^26^ In contrast, the microbial production of 1,8-cineole has been less extensively explored. To produce 1,8-cineole in *E. coli* we have used the bacterial cineole synthase from *Salvia fruticosa* (bCinS)^27^ with the highest reported titre of 120 mg L^−1^. Cineole production has been constrained by the promiscuity of cineole synthase and acetyl-CoA competition. A recent study addressed this by fusing CinS to farnesyl diphosphate synthase (IspA), creating substrate channelling and increasing the yield of 1,8-cineole to 1 g L^−1^.^28^

Here we introduce a new technology to produce VOCs (limonene and cineole) with microbes such as *E. coli*, called the Solid-State Platform (SSP). These highly volatile chemicals, terpenes, can be produced by recombinant *E. coli* inside the SSP. Limonene and cineole have previously been produced in *E. coli* using state-of-the-art techniques such as the two-phase method or *in situ* VOC capture. However, the SSP enables production and direct captures of limonene or cineole as vapour in the product container, eliminating the need to remove residual cell debris and growth medium.

## Experimental

### Molecular cloning

The gene of interest, bacterial cineole synthase (bCinS),^27^ was PCR-amplified using Phusion High-Fidelity DNA Polymerase (New England Biolabs) with primers containing overhangs for In-Fusion HD Cloning. The plasmid used for limonene production, pJBEI-6410 ^29^ was linearized by inverse PCR, to remove the limonene synthase (LimS), and serve as the recipient backbone for the bCinS (SI Fig. S1 and S2). All PCR products were purified using the Monarch DNA Gel Extraction Kit (New England Biolabs). Cloning was performed using the In-Fusion HD Cloning Kit (Takara Bio) following the manufacturer's protocol with primers containing with a 15–18 nt overhang. Single colonies were screened by colony PCR (OneTaq, Quick-Load, DNA Polymerase, from NEB) using one vector-specific primer and one bCinS specific primer. Positive clones were cultured in LB medium, and plasmids were extracted using the Monarch Plasmid Miniprep Kit (New England Biolabs, NEB). Insert verification was performed by primer walking with Sanger sequencing (Eurofins Genomics), confirming the sequence of plasmid pJBEI-6410-bCinS. As a control, pUC19 was used. Primers used in this study are listed in S Table. S1.

### Cell growth and solid-state platform (SSP)

**Fig. 1:**
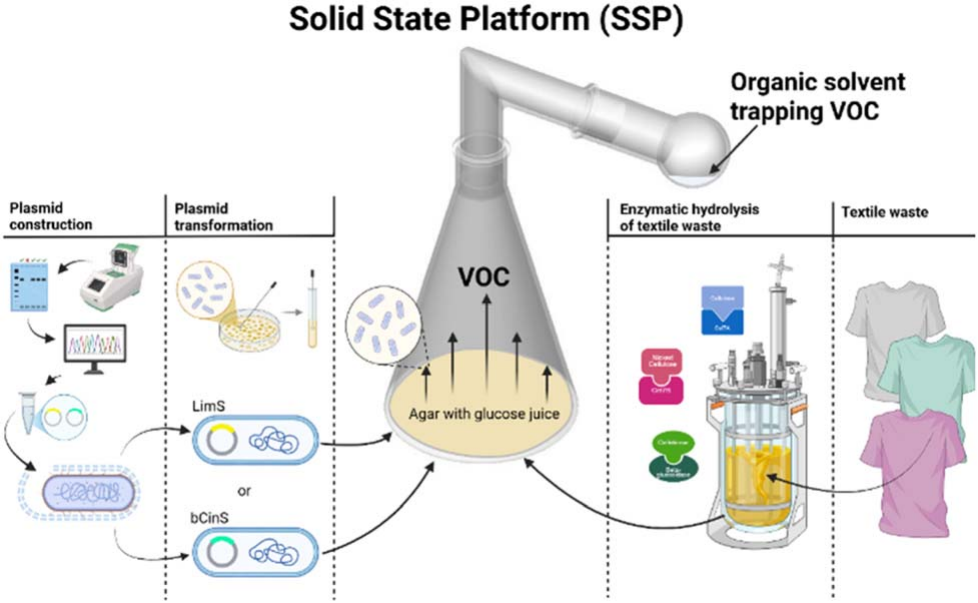
Schematic representation of the Solid-State Platform (SSP) with transformed *E. coli* harbouring pJBEI-6410 or pJBEI-6410-bCinS to produce VOCs from glucose or glucose juice.

**Fig. 2:**
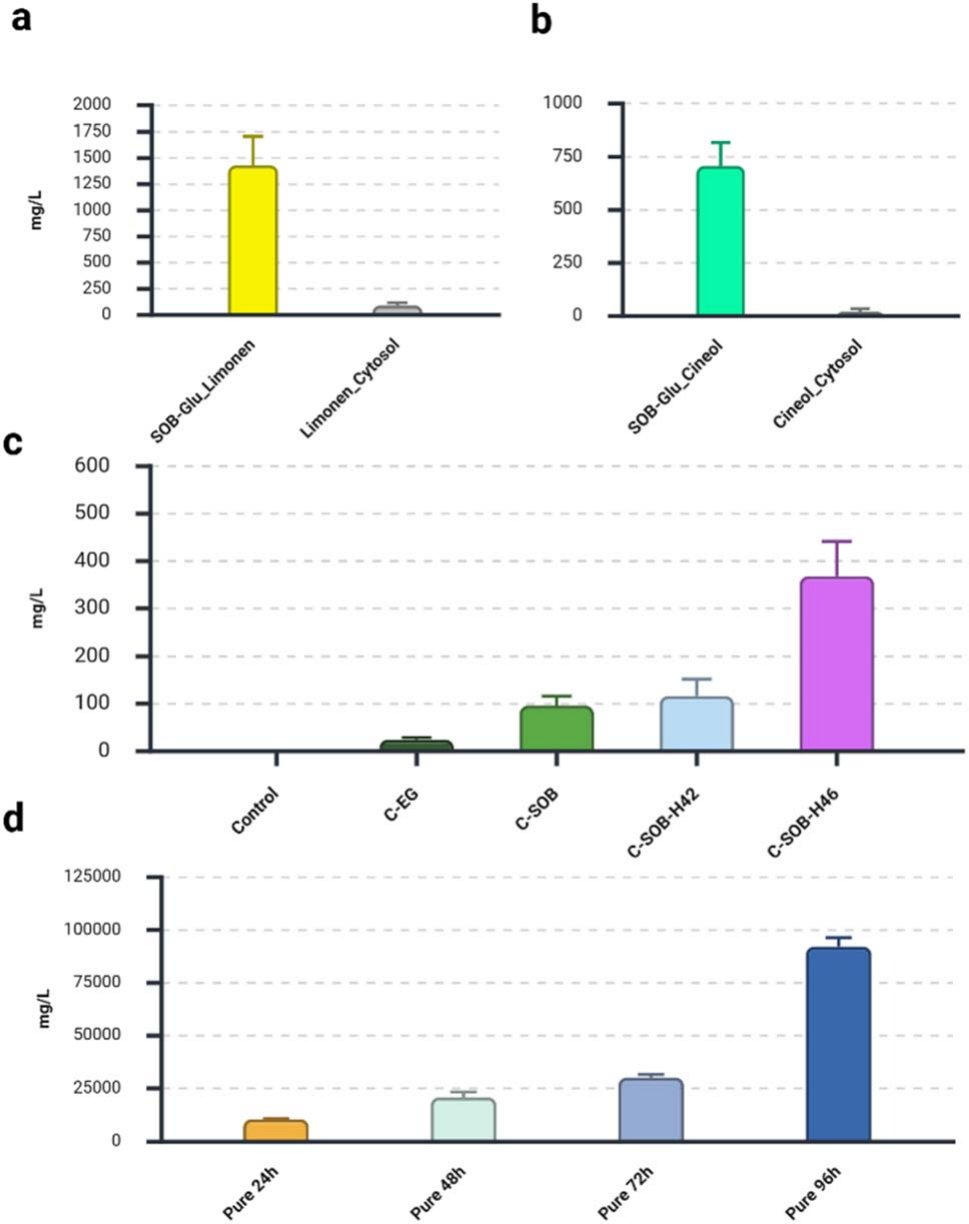
Graphic representation of the produced and captured terpenes measured by GC-MS after 96 h of fermentation. (a), limonene measured in SSP (yellow) and limonene in the cell cytosol (grey). (b), cineole measured in SSP (green/teal) and cineole measured cell cytosol. (c), cineol produced and captured on different carbon sources *via* SSP grown on different carbon sources (d), graphic representation of the captured cineole after 24 h, 48 h 72 h and 96 h in the product container of SSP using pure cineole as source of cineole emission. Error bars represent the standard deviation of three independent biological replicates.

*E. coli* TOP10 was used for all cloning procedures, and VOC production. Molecular cloning was performed in Luria-Bertani (LB) medium at 37 °C with agitation, 180 rpm. Ampicillin was added as required, 100 µg mL^−1^ during molecular cloning and for plasmid propagation. For cineole and limonene production, Super Optimal Broth (SOB) was solidified by adding 15 g L^−1^ agar and d-glucose (Glu), glucose juice (H42 and H46) or ethylene glycol (EG) was added at a final concentration of 0.4%, respectively. Solid media also contained 50 µg mL^−1^ ampicillin and 25 µM isopropyl-β-D-thiogalactoside (IPTG) to induce cineole or limonene production, respectively. A 1-liter Erlenmeyer flask with a ground glass joint, serving as a culture container in the Solid-State Platform (SSP) (Fig. 1 and SI S3a) was filled with 50 mL agar-containing medium, the appropriate carbon source and solidified at room temperature. A transformed single colony was picked and grown in 300 µl SOB medium for 3 h at 32 °C and then carefully spread on the full agar surface inside the SSP culture container using an extended hockey stick. Transformed *E. coli* contained either pJBEI-6410 to produce limonene or pJBEI-6410-bCinS to produce cineole or the control plasmid pUC19, respectively. The SSP was hermetically sealed with an appropriate ground glass linker (SI Fig. S3b) to connect the SSP culture container to the product container, a 50 mL round bulb with a grounded neck containing 1 mL of organic solvent dodecane (SI Fig. S3c). The culture container part of SSP was incubated for 96 h in water bath approximately 5 centimetres below water surface at 32 °C, while the room temperature was approx. 20 °C. After dismantling the SSP the remaining cells were scraped off the agar, resuspended in ethyl acetate, and lysed by vortexing to extract the remaining VOC trapped inside the cytosol (Fig. 2a and b). Experiments to determine the capture capacity of the SSP were performed with 5 mL of pure 1,8-cineole added to the culture container part of SSP, instead of the agar medium with *E. coli*.

### Enzymatic degradation

Two different textile waste streams were hydrolysed as previously described^25^ using the Cellic CTec2 from Novozymes. Substrate H42 represented post-industrial waste in the form of pure white, long fibres consisting of 100% of non-woven cotton, used as bedding fillers. Substrate H46, is post-consumer textile waste derived from apparel products (white working lab coats), made from polyester and cotton in a 65/35 ratio. All materials were shredded (pieces size 8 mm) before enzymatic treatment (SI Fig. S4).

The pretreated substrates H42 and H46 were suspended in 0.1 M sodium acetate buffer (pH 8) at a final concentration of 50 g textile per liter of buffer. The suspension was equilibrated at 50 °C with continuous agitation (250 rpm) for 30 min prior to enzyme addition. Enzymatic reactions were conducted in 0.1 M sodium acetate buffer (pH 8) at 50 °C in a total volume of 2,5 L containing the substrate and CelicTech2 enzyme blend. Aliquots (500 µL) were inactivated with 5 µL of 1 M NaOH to halt enzymatic activity and measure the concentration of glucose in the glucose juice from H42 and H46 by FTIR^14,25^ (SI Fig. S4). The resulting glucose juice concentration was adjusted to a final concentration of 0.4% in SSP production media.

### Sample preparation for qualitative and quantitative analysis

The VOCs produced by *E. coli* were trapped directly as vapor by the organic solvent (*n*-dodecane) that is present in the product container of the SSP. The dodecane containing the product was dried with MgSO_4_ to remove water completely. MgSO_4_ was removed by centrifugation for 5 min at 4 °C and 15 000 rpm. The dodecane containing the product was carefully decanted and diluted 1 : 1 with ethyl acetate. To control the volume of the injected sample, 0.1% sec-butylbenzene was used in the ethyl acetate mixture as an internal standard.

Prepared liquid samples were analysed by gas chromatography (GC-MS-QP 2010 Ultra, Shimadzu, Kyoto, Japan) equipped with a nonpolar column (Zebron TM ZB-5MSi, length 60 m, diameter 0.25 mm, film thickness 0.25 µm). Compounds were identified by MS (mass spectrometry; fragments were scanned in the range from 15 to 500 *m*/*z* and compared to the NIST 17 (National Institute of Standards and Technology) library) and quantified by flame ionization detection (FID).

The analysis was performed using the temperature-programmed method, where the temperature was first held at 90 °C for 2 minutes, then increased to 200 °C at a heating rate of 20 °C min^−1^, then increased to 230 °C at a heating rate of 5 °C min^−1^ and held at this temperature for 4 minutes, then increased to 310 °C at a heating rate of 10 °C min^−1^ and held at this temperature for 4.5 minutes. The injection volume was 0.5 µL and the split ratio was set to 80. The injector and FID detector temperature were maintained at 300 °C and 330 °C, respectively. Available standards limonene (Sigma-Aldrich, purity 99%) and cineole (Sigma-Aldrich, purity 99%) were used for quantification and 3–5 point calibration curves were prepared. Samples were analysed undiluted and/or diluted with ethyl acetate (Merck, purity ≥99.5%), 2-phenylbutane (Sigma-Aldrich, purity ≥99.0%), *n*-dodecane (Sigma-Aldrich, purity ≥99.0%) or *n*-hexane (LiChrosolv, purity ≥98.0%), as required. All standards for calibration curves were prepared as described above. Dilution factors were considered in the calculations when necessary.

## Results and discussion

The production of volatile organic compounds (VOCs) often terpenes such as cineole and limonene, by *E. coli* is typically performed in a liquid two-phase setup containing culture media and an organic solvent to trap the produced molecules. Here we introduce the Solid-State Platform (SSP), which allows direct trapping of VOCs (cineole or limonene) as vapor produced by engineered *E. coli*, without capturing cell debris or liquid media (Fig. 1).

Two different terpenes, 1,8-cineole and limonene were selected to demonstrate the overall concept that VOCs can be effectively produced *via* SSP. To produce limonene *E. coli* was transformed with the plasmid pJBEI-6410 ^29^ and induced with IPTG within the SSP. Approximately 1400 mg L^−1^ of limonene was captured when the production strain was grown for 96 hours on pure glucose (SOB-Glu) corresponding to a total of 1.4 mg captured in 1 mL of dodecane. The limonene that remained in the cell fraction was estimated to be less than 100 mg L^−1^, about 10% of the total limonene produced under this condition (Fig. 2a). Similarly, the plasmid pJBEI-6410-bCinS was used to produce cineole *via* SSP. Transformed cells were grown on d-glucose (SOB-Glu) and about 700 mg L^−1^ of cineole was captured in the SSP product container, which is about 0.7 mg total cineole per 1 mL of dodecane. In total only 20 mg L^−1^ of cineole, about 3%, remain inside the cells (Fig. 2b). These results demonstrate that limonene and cineole can be effectively produced and in case of cineole only a small fraction remains stuck inside the cells, showcasing the ability of SSP to produce cineole with minimal losses. The overall product profile of the synthases LimS^29^ and bCinS^27^ has been investigated previously.

The production of cineole *via* SSP, using four additional carbon sources was investigated: pure SOB (C-SOB), SOB with ethylene glycol (C-EG) and SOB with enzymatically hydrolysed textile waste (glucose juice) in H42 (C–H42) and H46 (C–H46). The efficiency of enzymatic hydrolysis on both textile waste streams, H42 and H46, was similar, yielding about 39 g of glucose per litter of buffer (SI Fig. S4). No cineole could be detected in the control culture transformed with pUC19. Interestingly only 25 mg L^−1^ cineole could be produced using EG, significantly less compared to the culture grown on SOB (Fig. 2c). Production on pure SOB was 95 mg L^−1^. The addition of glucose juice H42 resulted in 115 mg L^−1^ cineole production which is not significantly lower than the culture grown on SOB only. However, the addition of glucose juice H46 resulted in a significantly higher cineole titter of 350 mg L^−1^, which demonstrating its value for the production process. Interestingly, glucose juice H46 derived from textile waste that contained cellulose and PET, yet the production of cineole was higher compared to H42, which consists only of cellulose. Similar effects regarding the composition of enzymatically hydrolysed textile waste have also been observed also in conventional production of limonene using glucose juice from textile waste.^25^ These results demonstrate that enzymatically hydrolysed textile waste (glucose juice) can be utilized to produce cineole with the SSP.

Inside the culturing container *E. coli* grows on the bottom of the flask, which has a diameter of approximately 9.5 cm and is covered with 50 mL of solidified agar. The corresponding surface area of the agar using 50 mL of medium is roughly 70 cm^2^, which equates to 20 mg of limonene and 10 mg cineole produced per 1 cm^2^ of solidified medium. In comparison with conventional two-phase laboratory setups, SSP produced comparable amount of limonene^23^ and cineole^27^ per mL of organic solvent, dodecane. Only 50 mL of agar medium with the corresponding surface has been investigated to produce limonene and cineole, respectively in this study.

Since *E. coli* is highly resistant to cineole, tolerating up to 15 g L^−1^,^28^ the full capacity of SSP to capture cineole was investigated. To determine SSP's maximal cineole capture capacity, we used pure cineole instead of *E. coli* in the culture container as the source of cineole. The SSP was disassembled after 24 h, 48 h, 72 h, 96 h and directly measured by GC-MS (Fig. 2d). The capturing capacity of 1 mL dodecane in 24 h was remarkable, almost 10 g L^−1^, or a total of about 10 mg. After 48 h, around 20 g L^−1^ was captured and after 72 h, 30 g L^−1^ was captured, showing a linear increase over time. Finally, after 96 h, about 90 g L^−1^ was captured, which is about 90 mg or 10% w/v of dodecane. These results demonstrate the SSP's capacity to capture much more cineole in 96 h compared to what was produced with *E. coli*, 0.7 mg mL^−1^ with pure glucose and 0.35 mg mL^−1^ when using glucose juice H46.

Since the production level was far below the capacity that can be captured by SSP, future work will focus on optimizing the production strain, the culture container and fine-tuning the production conditions. The SSP strategy presented here demonstrates a scalable and sustainable platform for volatile terpene production. Unlike conventional bioreactors, SSP relies on simple laboratory glassware, with dodecane ensuring efficient vapor trapping and product recovery. The system eliminates the need for complex gaskets or filters, reducing operational costs and making it accessible for broader applications. The results validate the robustness of SSP for VOC biosynthesis, with glucose juice derived from textile waste offering a renewable and sustainable alternative to pure glucose (Table 1).

**Table 1:** A partial list of factors to consider when comparing SSP with previously known technology^25^

Risk type	Metric
Energy usage	No losses due to decanting of solvent from media and no centrifugation needed to separate cells/media
Mass of waste	Cells are cultured in inert glass containers that can be autoclaved and reused. Products/solvent is not contaminated with cells
Water consumption	Higher water consumption by SSP per mg of produced VOC, compared to previously known technologies^25^

## Conclusions

SSP can be used to produce and capture many VOCs from different emission sources while utilizing all applicable substrates. Using pure glucose, the final production of VOCs- limonene and cineole-is comparable^23,27^ to the state-of-the-art two-phase VOC capture approaches. In a single run of SSP we produced 1.4 mg mL^−1^ of limonene, which is a total of 1.4 mg per SSP experiment and 0.7 mg mL^−1^ of cineol equivalent to a total of about 0.7 mg of cineole per run. Future studies should focus on improving the culture container by increasing the surface to volume ratio of the solidified agar. Additionally metabolic pathway engineering, genomic modifications or integration of the full pathway should be performed in *E. coli*^30^ to further optimize cineole production and move closer to the capturing capacity of SSP. However, implementing new technologies requires addressing systemic barriers, including inadequate recycling infrastructure, limited public awareness and insufficient policy frameworks. These barriers must be overcome with coordinated efforts for scalable and sustainable solutions, ensuring that textiles move from waste to resource in line with circular economy principles. Future directions may leverage on AI-driven solutions such as smart AI controlled fermentation, AI enzyme design, and AI pathway optimizations to further increase titters and diversify products.

## Author contributions

Z. Z. and A. L. Have conceived this study. Z. Z. Coordinated the project, performed the molecular cloning, led the initial production experiments and quantification with GC-MS. The SSP experiments were performed by V. B., including enzymatic treatment of waste materials, while B. H. Quantified the resulting product with GC-MS under the supervision of M. G and B. L. The textile waste was selected M. P. All the authors interpreted the results. Z. Z. wrote the paper, with contributions from all authors.

## Conflicts of interest

Z. Z. and A. L. filed a patent application under the number EP23151430.8 published under the number EP4400574.

## Supplementary Material

RA-016-D5RA06351E-s001

## Data Availability

All data obtained in this study are included in this published article and its supplementary information (SI). However, the raw data analysed are available on request from the corresponding author. Supplementary information: PCR primers, bCinS sequence, pictures of SSP, enzymatic hydrolysis: Add LINK. See DOI: https://doi.org/10.1039/d5ra06351e.
