# Supplementary material for: Solid-state platform (SSP) to produce terpenes from enzymatically treated textile waste with E. coli
Source: RSC Adv. 2026 Jan 14;16(4):3163–8. doi: 10.1039/d5ra06351e (PMC12801246; doi:10.1039/d5ra06351e)
Supplement: RA-016-D5RA06351E-s001 [file RA-016-D5RA06351E-s001.pdf]

Supplementary Information (SI) for RSC Advances.
This journal is © The Royal Society of Chemistry 2025

Supplementary material

Solid-State Platform (SSP) to produce terpenes from enzymatically treated textile waste with *E. coli*

Žiga Zebec[1,2,3,*], Vid K. Bučar[1,], Brigita Hočevar[,4], Mojca Poberžnik[1], Miha Grilc[4], Blaž Likozar[4] and Aleksandra Lobnik[1,2]

1 – Institute for sensors and environmental protection, IOS d.o.o, Maribor, Slovenia
2 – University of Maribor, Faculty of Mechanical engineering, Maribor, Slovenia
3 – **Current affiliation**, Institute of informational science Maribor, IZUM, Slovenia
4 – Chemical Institute, Ljubljana, Slovenia.
* - Correspondence.

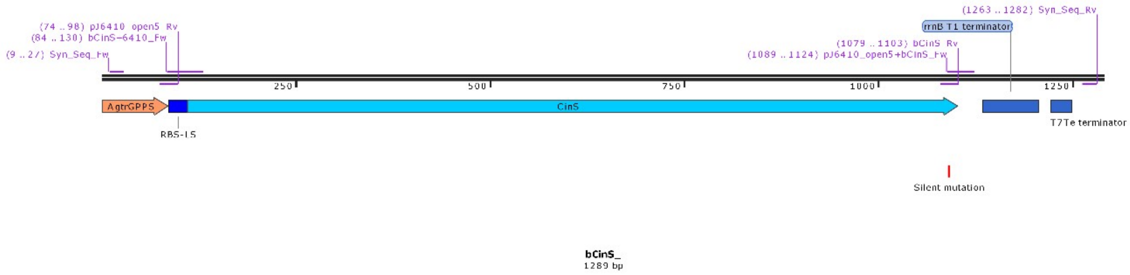

Supplementary Figure 1. Schematic representation of bCinS and the primers used for cloning and sequencing.

cgaagggtgaactgtcctgcttcgacccagttaaagccgctccgctgctgggcctggcagactacgtggcatttcgtcagaattaaggatcttttaag
aaggagatatacATGCCTGCAGGTCATGAAGAATTTGATATTCCGTTTCCGAGCCGTGTTAATCC
GTTTCATGCACGTGCCGAAGATCGTCATGTTGCATGGATGCGTGCAATGGGTCTGATTAC
CGGTGATGCAGCAGAAGCAACCTATCGTCGTTGGAGTCCGGCAAAAGTTGGTGCACGTTG
GTTTTATCTGGCACAGGGTGAAGATCTGGATCTGGGTTGTGATATTTTTGGTTGGTTTTTC
GCCTATGATGATCACTTTGATGGTCCGACCGGCACCGATCCGCGTCAGACCGCAGCATTT
GTTAATCGTACCGTTGCAATGCTGGATCCGCGTGCCGATCCGACCGGTGAACATCCGCTG
AATATTGCATTTCATGATCTGTGGCAGCGTGAAAGCGCACCGATGAGTCCGCTGTGGCAA
CGTCGTGCAGTTGATCATTGGACCCAGTATCTGACCGCACATATTACCGAAGCCACCAAT
CGTACCCGTCATACCAGCCCGACCATTGCAGATTATCTGGAACTGCGTCATCGTACCGGT
TTTATGCCTCCGCTGCTGGATCTGATTGAACGTGTTTGGCGTGCAGAAATTCCGGCACCGG
TTTATACCACACCGGAAGTTCAGACCCTGCTGCATACCACCAATCAGAATATTAACATTG
TGAACGATGTGCTGAGCCTGGAAAAAGAAGCACACGGCGATCCGCATAATCTGGTT
CTGGTTATTCAGCATGAACGTCAGAGCACCCGTCAGCAGGCACTGGCAACCGCACGTCGT
ATGATTGATGAATGGACCGATACCTTTATTCGTACCGAACCGCGTCTGCCTGCACTGTGTG
GTCGTCTGGGTATTCCGCTGGCAGATCGTACCAGCCTGTATACCGCAGTTGAAGGTATGC
GTGCAGCCATTCGTGGTAATTATGATTGGTGTGCCGAAACCAATCGTTATGCAGTTCATC

GTCCGACAGGTACAGGTCGTGCAACaACCCCGTGGTAAggatccaaactcgagtaaggatctccaggcatcaa ataaaacgaaaggctcagtcgaaagactgggcctttcgttttatctgttgtttgtcggtgaacgctctctactagagtcacactggctcaccttcgggtg ggcctttctgcgtttatacctagggatatattccgcttcctcgctcactga

Supplementary Figure 2. Full sequence of bCinS (teal color, coding sequence) with upstream and downstream region.

Supplementary Table 1. Primers used for molecular cloning.

**Primer name**	**5' - 3' sequence**	**Use**
pJ6410_open5_Fw	ACAACCCCGTGGTAAGGATCCAAACTCGAGTAAGGA	Linearization of Plasmid
pJ6410_open5+bCinS_Rv	acaaccccgtggTAAGGATCCAAACTCGAGTAAGGA	Linearization of Plasmid
bCinS+6410_Fw	taaggatcttttaagaaggagatatacATGCCTGCAGGTCATGAAGA	Amplification of bCins
bCinS_Rv	TTACCACGGGGTTGTTGCACGACCT	Amplification of bCins
Syn_Seq_Fw	gaactgtcctgcttcgacc	Sanger sequencing
Syn_Seq_Rv	gcgaggaagcggaatatatc	Sanger sequencing

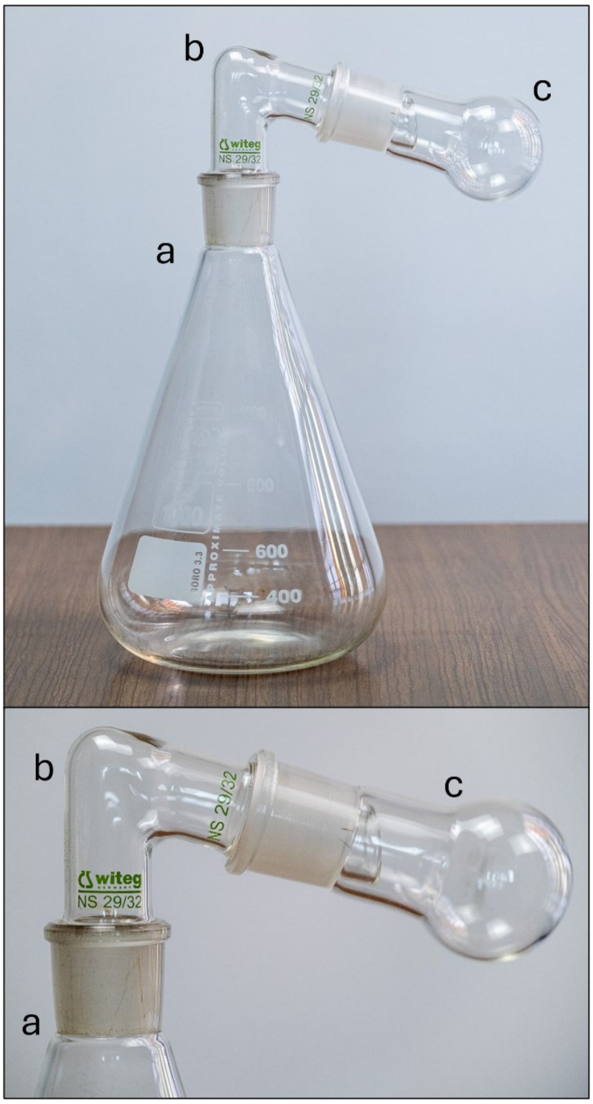

Supplementary Figure 3. Components of the SSP. **a,** Culture container, 1 Liter Erlenmeyer flask with ground glass joint size NS 29/32. **b,** Grounded glass linker with NS 29/32 connector on both sides. **c,** Product container, a 50 ml round bulb with a grounded neck with size NS 29/32.

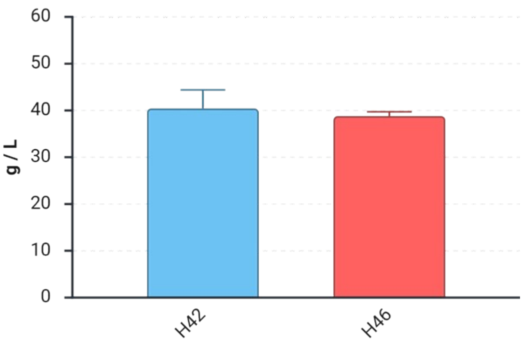

Supplementary Figure 4. Enzymatic hydrolysis of textile waste streams H42 (blue) and H46 (red), measured by FTIR. The columns represent the average of three technical replicates with error bars representing standard deviation (SD).
